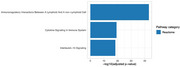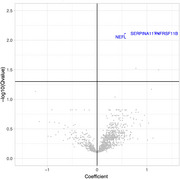# Plasma proteomic signatures of cerebrovascular disease

**DOI:** 10.1002/alz70856_103352

**Published:** 2025-12-26

**Authors:** Ming Ann Sim, Hyung Won Choi, Yuan Cai, Eugene Tan, Saima Hilal, Arthur Mark Richards, Christopher Chen

**Affiliations:** ^1^ National University of Singapore, Singapore, Singapore, Singapore; ^2^ National University Hospital, Singapore, Singapore, Singapore; ^3^ Lau Tat‐chuen Research Centre of Brain Degenerative Diseases in Chinese, Gerald Choa Neuroscience Institute, Therese Pei Fong Chow Research Centre for Prevention of Dementia, The Chinese University of Hong Kong, Prince of Wales Hospital, Hong Kong SAR, Hong Kong; ^4^ National University Heart Centre Singapore, Singapore, Singapore, Singapore; ^5^ Erasmus MC, Rotterdam, Netherlands; ^6^ Christchurch Heart Institute, University of Otago, New Zealand, Christchurch, New Zealand; ^7^ Memory, Ageing and Cognition Centre, National University Health System, Singapore, Singapore

## Abstract

**Background:**

Cerebrovascular disease (CeVD) is a clinically significant disease entity, known to be associated with increased risk of cognitive decline [1]. However, the lack of validated blood biomarkers for CeVD, limits advances in detection and intervention. We thus aim to identify the plasma protein signatures of CeVD, its phenotypes, and progression.

**Method:**

A prospective Singaporean memory clinic cohort was followed‐up for 4 years. Baseline and 2‐yearly brain magnetic resonance imaging (MRI) scans were performed. Significant CeVD was defined as the presence of cortical infarcts, and/or ≥ 2 lacunes, and/or ARWMC (age related white matter changes) score of ≥ 8.

The burden of CeVD lesions by phenotypes (lacunes, cortical infarcts, white matter hyperintensity volume (WMHv) and cerebral microbleeds (CMBs)) was then evaluated at baseline. The longitudinal progression of WMHv was evaluated using volumetric measurements of WMHv on 2‐yearly MRI brain scans.

The Olink Explore platform was used to profile 1536 plasma proteins at baseline. Their associations with cross‐sectional and longitudinal CSVD imaging markers were evaluated using multivariable regression models, accounting for multiple testing correction (target FDR 5%).

**Result:**

Of 508 included subjects (mean age 72.8±7.8 years, 56.1% female, 72% hypertensive), 53% had significant CeVD.

We identified 345 proteins associated with significant CeVD, implicated in biological pathways related to inflammation, immune dysregulation, and cell adhesion (top 3 over‐represented biological pathways presented in Figure 1).

We identified distinct and shared proteins associated with lacunes, CMBs, cortical infarcts, and WMHv. The most significantly dysregulated protein for CMBs was CHRDL1 (RR 5.30, 95% C.I. 2.93‐9.61, q‐value<0.05). The most significant protein associated with cortical infarcts was NTproBNP (RR 1.38, 95% C.I. 1.23‐1.54, q‐value<0.05). NFL (RR 1.44, 95% C.I. 1.44, 95% C.I. 1.29‐1.60, q‐value<0.05) was most significantly associated with lacune burden, and WMHv (β 1.15, 95% C.I. 0.76‐1.54, q‐value<0.05).

In a separate analysis of 493 subjects with longitudinal WMHv data, the most significant proteins of longitudinal WMHv progression included TNRSF11B, SERPINA11 and NFL (Figure 2).

**Conclusion:**

We report plasma proteomic signatures for CeVD. Further studies are required to evaluate the mechanistic underpinnings of these plasma biomarkers as therapeutic targets for CeVD pathology.

**References**

1. Elahi, et al. Stroke 2023.